# (*E*)-2,4-Di­amino-5-{7-[(4-chloro­phen­yl)diazen­yl]-3,3-dimethyl-1-oxo-2,3,4,9-tetra­hydro-1*H*-xanthen-9-yl}-6-oxo-1,6-di­hydro­pyridine-3-carbo­nitrile di­methyl­formamide monosolvate

**DOI:** 10.1107/S2414314625009393

**Published:** 2025-11-06

**Authors:** Galal H. Elgemeie, Nadia H. Metwally, El-shimaa S. M. Abd Al-latif, Peter G. Jones

**Affiliations:** a Chemistry Department, Faculty of Science, Helwan University, Cairo, Egypt; b Chemistry Department, Faculty of Science, Cairo University, Giza, Egypt; c Institut für Anorganische und Analytische Chemie, Technische Universität Braunschweig, Hagenring 30, D-38106 Braunschweig, Germany; Goethe-Universität Frankfurt, Germany

**Keywords:** diazene, xanthene, hydrogen bond, crystal structure

## Abstract

In the title compound, C_27_H_23_ClN_6_O_3_·C_3_H_7_NO, much of the mol­ecule is approximately planar, excluding the pyridinic ring, which is almost perpendicular to this plane, and the *sp*^3^ atoms of the modified xanthene system. The diazene group is *E*-configured. In the extended structure, two hydrogen bonds of the type N—H⋯O and one N—H⋯Cl combine to form a layer structure parallel to (111). The solvent is severely disordered and this necessitated the use of SQUEEZE for a reliable refinement.

## Abstract 2

In the title compound, much of the mol­ecule (excluding the pyridinic ring) is approximately planar. In the extended structure, various hydrogen bonds combine to form a layer structure parallel to (111).

## Graphical abstract

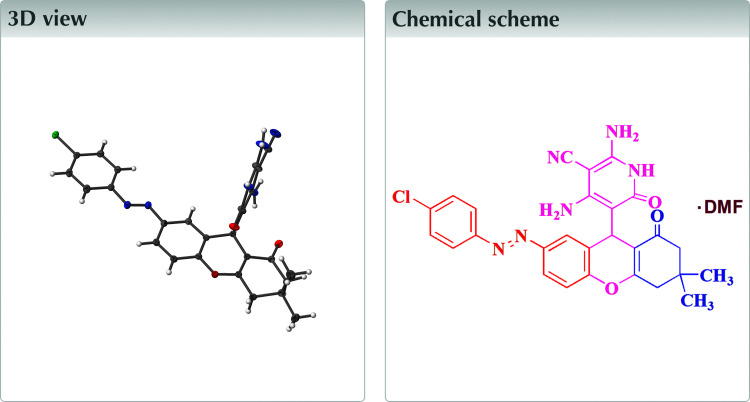

## Structure description

In a variety of chemical processes, activated nitriles, which involve active methyl­ene groups, are used for the synthesis of heterocyclic, pharmaceutically significant compounds (Wang *et al.*, 2016 ▸; Fleming & Wang, 2003 ▸; Zhang *et al.*, 2023 ▸; Abu-Zaied *et al.*, 2024*a* ▸,*b* ▸; Zhang *et al.*, 2019 ▸). Using such nitriles as starting materials, we have published a number of new approaches for the synthesis of heterocycles (Elgemeie *et al.*, 1998*a* ▸,*b* ▸, 2010 ▸). Using dimedone as the starting material, we and others have continued this work by synthesizing a number of condensed carbocyclic pyridines and carbocyclic pyrans (Hebishy *et al.*, 2022 ▸, 2023 ▸; Tu *et al.*, 2014 ▸). The current study describes a one-pot synthesis of a tetra­hydroxanthene derivative by the reaction of dimedone with enamino nitriles and *o*-hy­droxy aromatic aldehydes.

**Figure 1:**
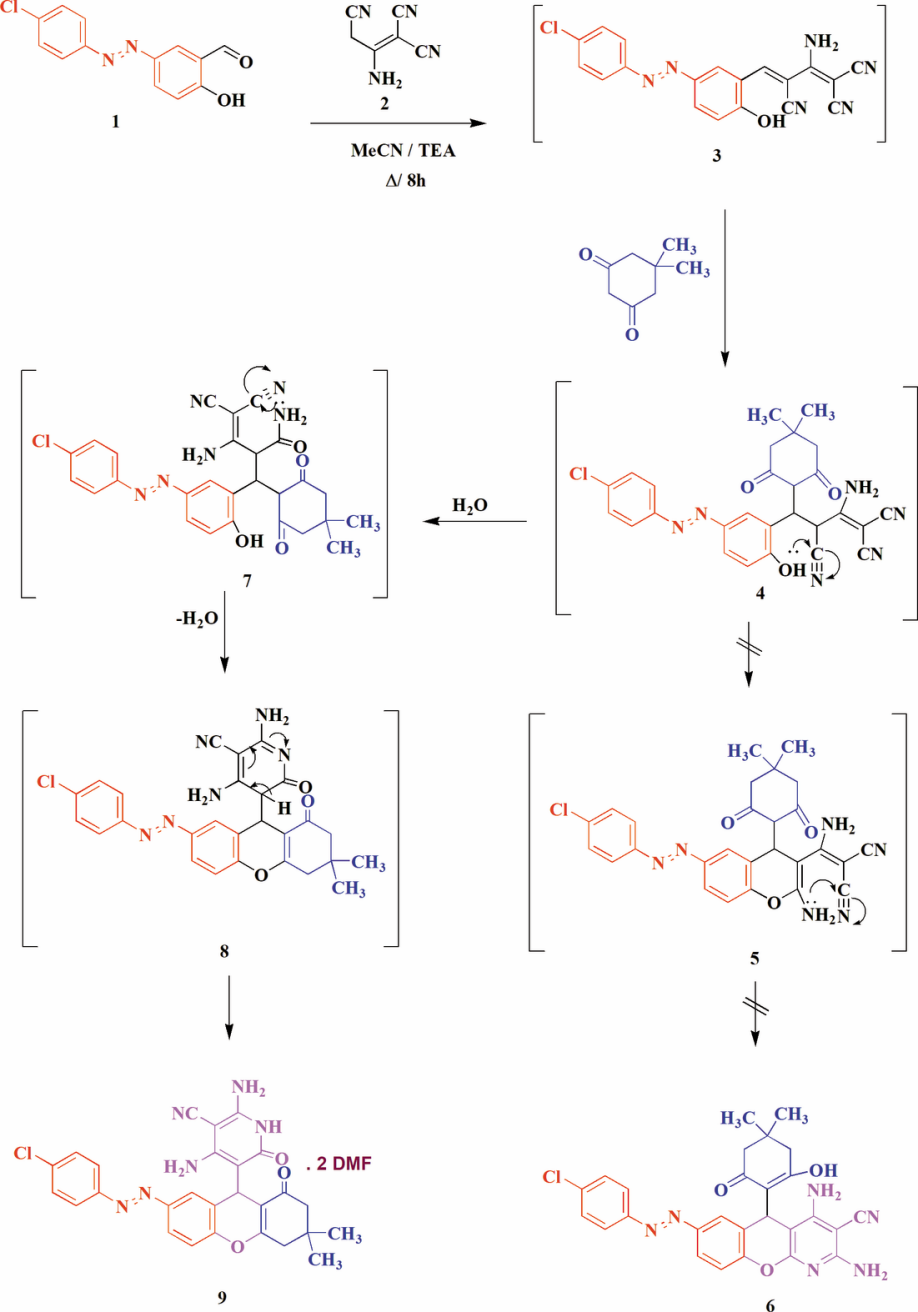
The reaction scheme and proposed mechanism for the formation of **9**.

It was found (Fig. 1 ▸) that dimedone reacted with (4-chloro­phen­yl)diazenyl-2-hy­droxy­benzaldehyde) (**1**) and 2-amino­prop-1-ene-1,1,3-tricarbo­nitrile (**2**) in refluxing aceto­nitrile containing catalytic amounts of tri­methyl­amine to give the condensation product (*E*)-5-{7-[(4-chloro­phen­yl)diazen­yl]-2,3,4,9-tetra­hydro-3,3-dimethyl-1-oxo-1*H*-xanthen-9-yl}-2,4-di­amino-1,6-di­hydro-6-oxo­pyridine-3-carb­o­nitrile (**9**). The structure of **9** was suggested by elemental analysis and spectroscopic studies (^1^H NMR, IR and MS). As a mechanism we propose a condensation reaction that consists of an initial Michael addition of the methyl­ene group of the dimedone to the double bond of inter­mediate **3** to give a further inter­mediate **4**, which then cyclizes to give the tetra­hydroxanthene structure **9** rather than the alternative cyclization leading to the chromeno[2,3-*b*]pyridine structure **6**. The same reaction has been carried out, under the same reaction conditions, by other researchers, who however stated that they obtained structure **6** as the sole product, but no X-ray single-crystal studies were performed (Vereshchagin *et al.*, 2017 ▸; Ryzhkova *et al.*, 2022 ▸). In order to establish the structure of the compound unambiguously, the crystal structure of **9** was determined and is presented here.

**Figure 2:**
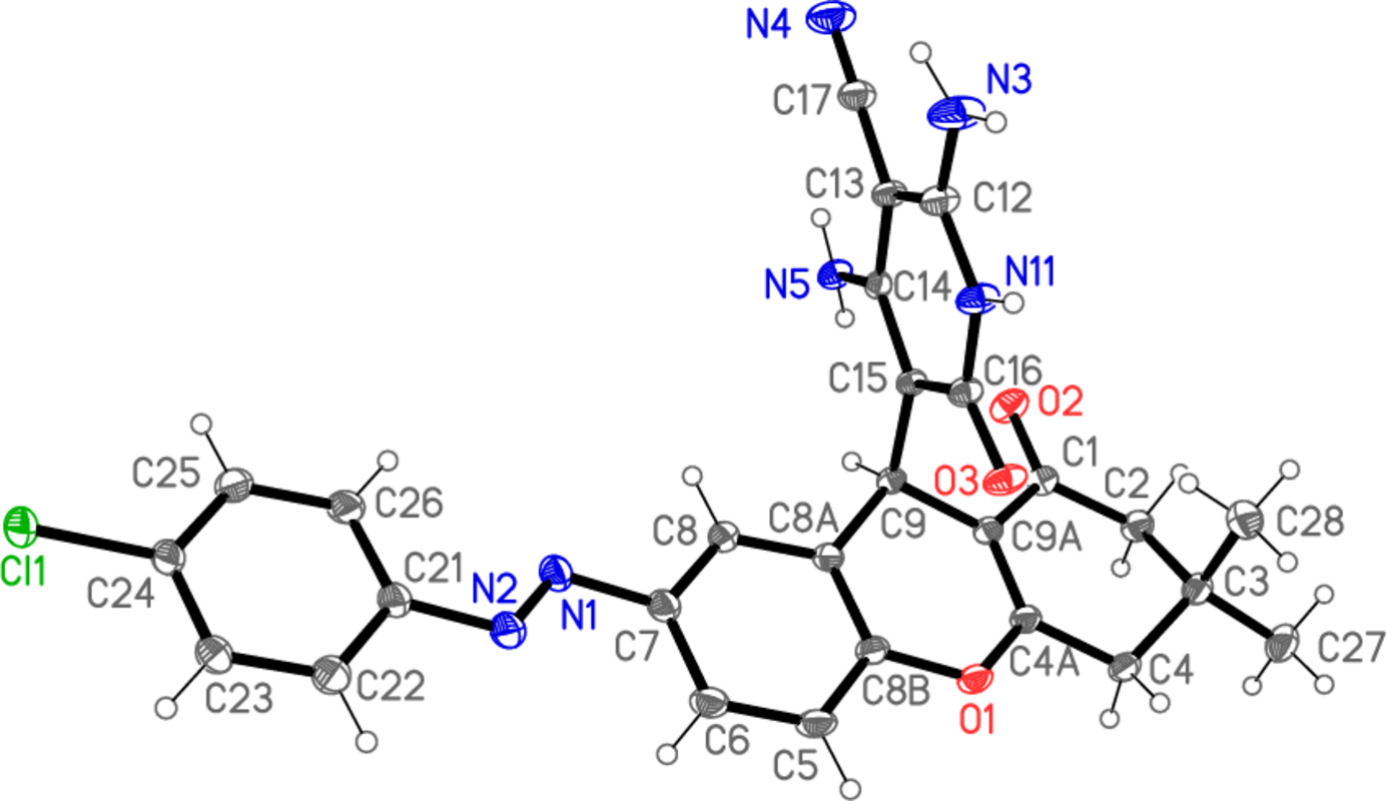
The mol­ecule of compound **9** in the crystal (excluding the severely disordered solvent). Ellipsoids are drawn at the 50% probability level.

**Figure 3:**
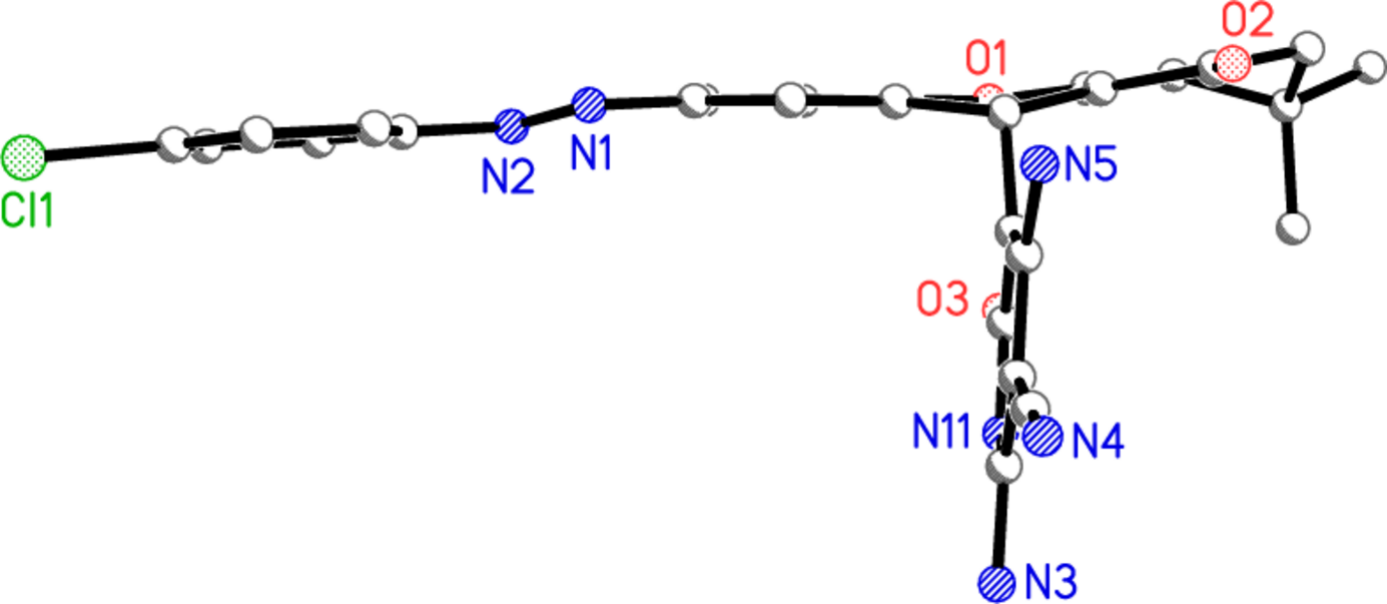
Side view of the mol­ecule of **9** (excluding hydrogen atoms); radii are arbitrary.

The structure of compound **9** (excluding solvent, see *Refinement details*) is shown in Figs. 2 ▸ and 3 ▸. Mol­ecular dimensions, a brief selection of which are given in Table 1 ▸, may be regarded as normal. Despite the presence of *sp*^3^ carbon atoms and the possibility of rotation about the C—N bonds to the *E*-configured diazene group, much of the mol­ecule is approximately planar (Fig. 3 ▸); excluding the pyridinic ring and the atoms C3, C27 and C28, the r.m.s. deviation from the best plane is 0.10 Å. The pyridinic ring (including substituents) has an r.m.s. deviation of 0.03 Å and subtends an inter­planar angle of 88.66 (2)° with the main plane. The intra­molecular hydrogen bond H051⋯O2, not drawn explicitly in Fig. 2 ▸ for reasons of clarity, is part of a three-centre system (Table 2 ▸).

**Figure 4:**
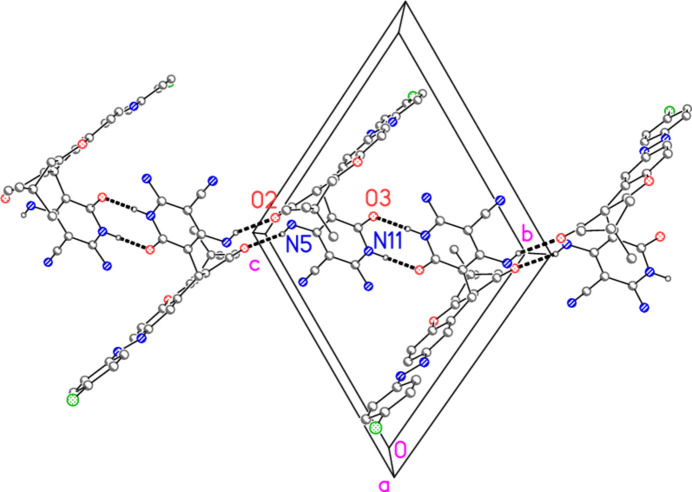
Packing of compound **9** viewed parallel to the *a* axis (thick dashed lines indicate hydrogen bonds; atoms of the asymmetric unit are numbered). Hydrogen atoms not involved in the hydrogen bonds N11—H011⋯O3′ and N5—H052⋯O2′ are omitted. Labels indicate atoms of the asymmetric unit.

**Figure 5:**
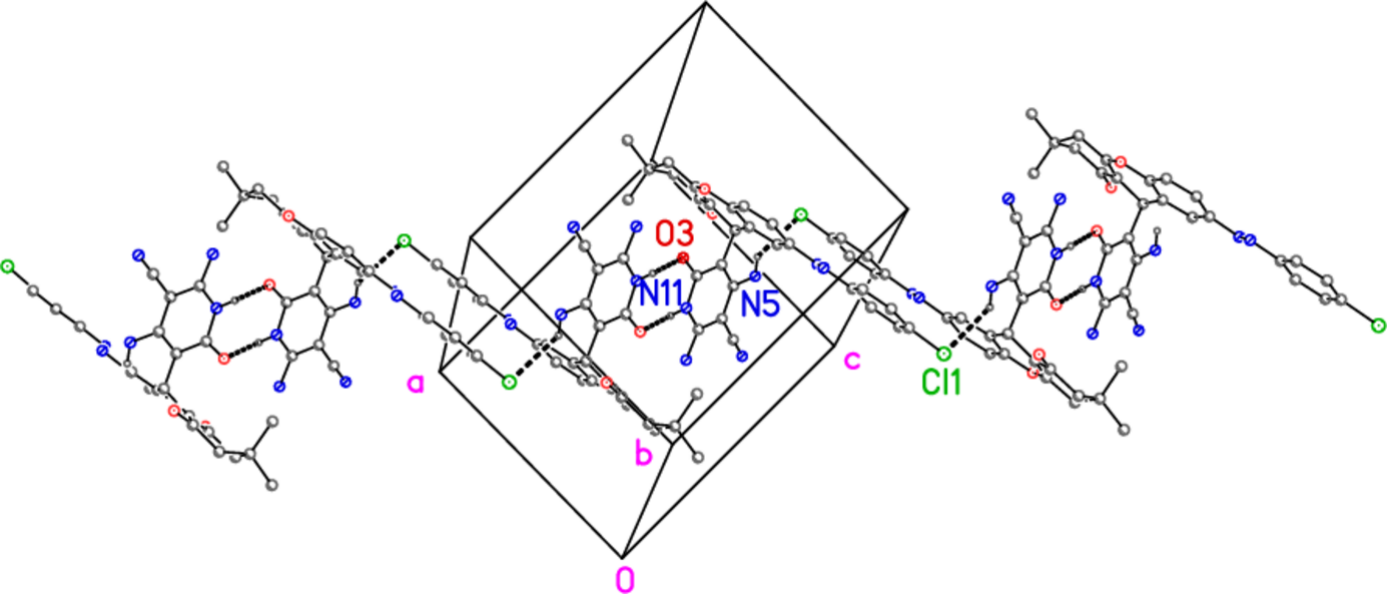
Packing of compound **9** viewed perpendicular to the *ac* plane (thick dashed lines indicate hydrogen bonds; atoms of the asymmetric unit are numbered). Hydrogen atoms not involved in the hydrogen bonds N11—H011⋯O3′ and N5—H051⋯Cl1′ are omitted. Labels indicate atoms of the asymmetric unit.

**Figure 6:**
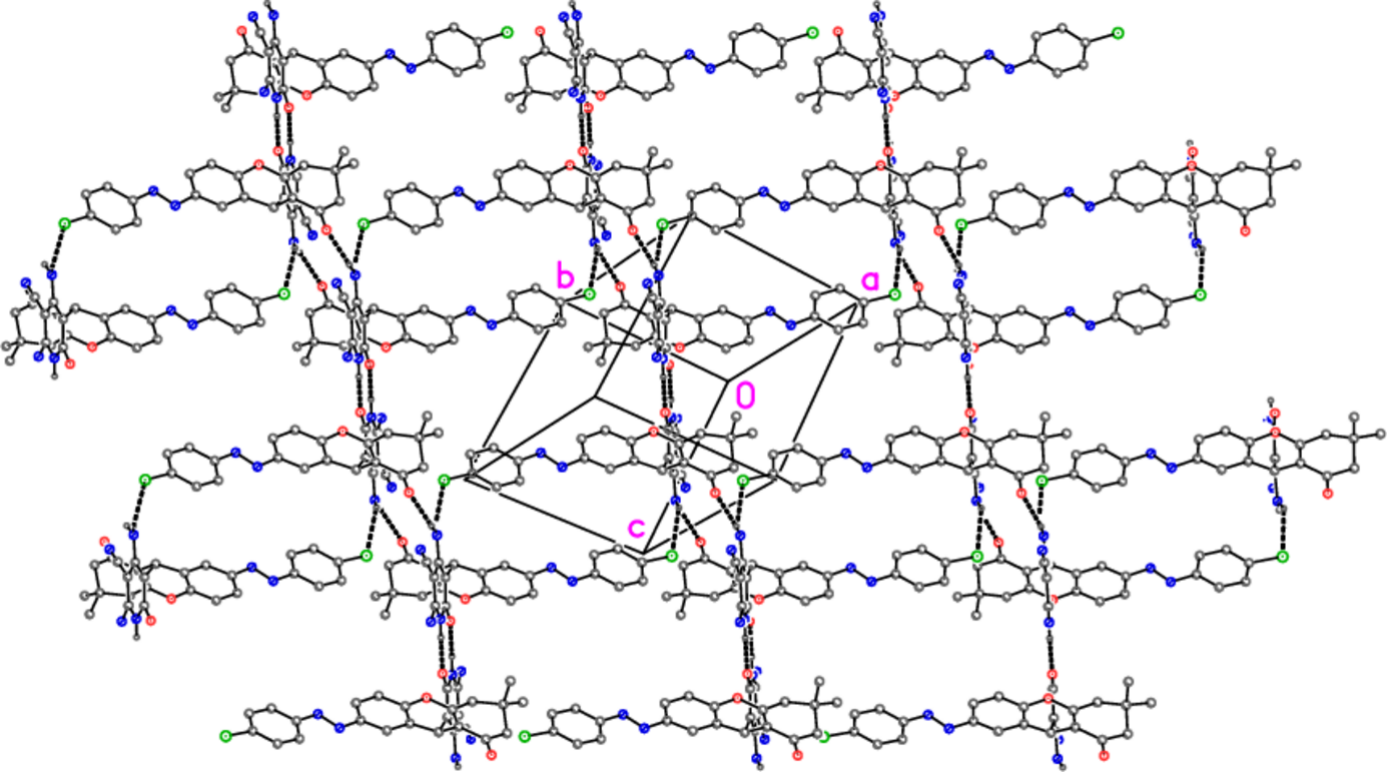
The layer structure of compound **9** viewed perpendicular to (111) (thick dashed lines indicate hydrogen bonds). Hydrogen atoms not involved in hydrogen bonds are omitted.

The mol­ecular packing may be analysed in terms of hydrogen bonds (Table 3 ▸). The hydrogen bonds N11—H011⋯O3′ and N5—H052⋯O2′ combine to form a one-dimensional array propagating in the [01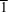] direction (Fig. 4 ▸), whereas N11—H011⋯O3′ and N5—H051⋯Cl1′ form a one-dimensional array parallel to [10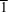] (Fig. 5 ▸). The zone law then suggests that the layer structure formed by all three hydrogen bonds should be parallel to (111), which is indeed the case (Fig. 6 ▸). We note that the potential hydrogen bond donors at N3 do not appear to form hydrogen bonds; in fact there are short contacts between these hydrogen atoms and the difference peaks arising from the severely disordered solvent.

A search of the Cambridge Database (Version 2025.1.1; Groom *et al.*, 2016 ▸) using the routine CONQUEST (Bruno *et al.*, 2002 ▸) found no other examples of a similarly modified xanthene derivative either with a nitro­gen substituent at C7 or a nitro­gen heterocycle at C9.

## Synthesis and crystallization

A mixture of 4-(chloro­phenyl­diazen­yl)-2-hy­droxy­benz­aldehyde **1** (2.6 g, 0.01 mmol), 2-amino­prop-1-ene-1,1,3-tricarbo­nitrile **2** (1.32 g, 0.01 mmol), 5,5-di­methyl­cyclo­hexane-1,3-dione (‘dimedone’, 1.4 g, 0.01 mmol) and few drops of tri­methyl­amine in aceto­nitrile (30 ml) was refluxed for 8 h. After cooling, the precipitate of compound **9** was collected by filtration and recrystallized from dimethylformamide (DMF) as large orange blocks. Yield 4.00 g (78%). For X-ray measurements, an irregular single crystalline fragment of suitable dimensions was cut from a larger block.

M.p.: above 573 K. ^1^H NMR (400 MHz, DMSO-*d*_6_): *δ*H = 0.98 (*s*, 3H, CH_3_), 1.06 (*s*, 3H, CH_3_), 2.07 (*d*, 1H, *J* = 16.4 Hz, CH_2_), 2.33 (*d*, 1H, *J* = 16.14 Hz, CH_2_), 2.40–2.59 (*m*, 2H, CH_2_), 4.94 (*s*, 1H, pyran-H), 6.35 (*s*, 2H, NH_2_), 6.47 (*s*, 2H, NH_2_), 7.14 (*d*, 1H, *J* = 8.64 Hz, Ar—H), 7.55 (*s*, 1H, Ar—H), 7.60 (*d*, 2H, *J* = 8.6 Hz, Ar—H), 7.67–7.69 (*m*, 1H, Ar—H), 7.85 (*d*, 2H, *J* = 8.6 Hz, Ar—H), 9.67 (*s*,1*H*, NH) p.p.m.. ^13^C NMR (100 MHz, DMSO-*d*_6_): *δ*C = 26.82, 27.60, 29.48, 31.24, 32.21, 50.82, 62.50, 99.39, 110.75, 116.73, 117.43, 121.98, 124.17, 124.55, 127.23, 129.95, 135.99, 148.50, 151.02, 153.27, 153.38, 155.05, 160.20, 162.77, 165.10, 196.91. Analysis calculated for C_27_H_23_ClN_6_O_3_ (514.96): C 62.97, H 4.50, Cl 6.88, N 16.32. Found: C 62.80, H 4.69, N 16.08%.

## Refinement

Crystal data, data collection and structure refinement details are summarized in Table 3 ▸.

Hydrogen atoms bonded to nitro­gen were refined with an N—H distance restraint (SADI) for the NH_2_ groups. Methyl groups were refined as idealized rigid groups allowed to rotate but not tip (‘AFIX 137’), with C—H = 0.98, H—C—H = 109.5°. Other hydrogen atoms were included using a riding model starting from calculated positions (C—H_methine_ = 1.00, *C*—H_methyl­ene_ = 0.99 Å). The *U*_iso_(H) values were fixed for methyl groups at 1.5 × *U*_eq_, and for other H atoms at 1.2 × *U*_eq_ of the parent carbon atoms. Three badly-fitting reflections (deviations > 7.5σ) were omitted from the refinement. The weighting parameters *a* and *b* (Sheldrick, 2015*b* ▸) oscillated over a small range.

A region of residual electron density around the inversion centre at (0, 0, 1/2) was tentatively inter­preted as several overlapping (and thus partially occupied) DMF sites (DMF was used for the recrystallization). One clear DMF position could be refined (with occupation 0.58) but the remaining difference peaks could not be inter­preted satisfactorily; no suitable model of disordered DMF was found. It is possible that some other solvent, perhaps remaining from the synthesis, may be involved. The routine SQUEEZE (as implemented in the program system *PLATON*; Spek, 2020 ▸) was used to remove mathematically the effects of the disordered solvent. The electron content of the void was estimated as 98, corresponding to two DMF molecules per void (and thus per cell) and one DMF per asymmetric unit. This content was used to calculate the formula weight and other related qu­anti­ties, but should be inter­preted with caution. The number of parameters in the refinement was adjusted upwards by 55 (recommended by the SQUEEZE routine; command ‘L.S. 6 0 55’) to allow for the solvent parameters when calculating su’s. The use of SQUEEZE causes a long series of ‘G ALERTS’ when the CIF file is analysed by checkCIF.

## Supplementary Material

Crystal structure: contains datablock(s) I, global. DOI: 10.1107/S2414314625009393/bt4183sup1.cif

Structure factors: contains datablock(s) I. DOI: 10.1107/S2414314625009393/bt4183Isup2.hkl

Supporting information file. DOI: 10.1107/S2414314625009393/bt4183Isup3.cml

CCDC reference: 2497635

Additional supporting information:  crystallographic information; 3D view; checkCIF report

## Figures and Tables

**Table 1:** Selected geometric parameters (Å, °)

N1—N2	1.2572 (13)	N11—C16	1.3875 (12)
N1—C7	1.4217 (13)	C12—N3	1.3517 (14)
N2—C21	1.4224 (14)	C14—N5	1.3489 (12)
N11—C12	1.3483 (13)		
			
N2—N1—C7	112.74 (9)	C12—N11—C16	124.32 (8)
N1—N2—C21	114.76 (9)		
			
C7—N1—N2—C21	−179.90 (8)	C9*A*—C9—C15—C16	−64.70 (11)
C9*A*—C9—C15—C14	118.20 (10)	C8*A*—C9—C15—C16	57.63 (11)
C8*A*—C9—C15—C14	−119.47 (10)		

**Table 2:** Hydrogen-bond geometry (Å, °)

*D*—H⋯*A*	*D*—H	H⋯*A*	*D*⋯*A*	*D*—H⋯*A*
N11—H011⋯O3^i^	0.898 (17)	1.758 (17)	2.6558 (11)	178.7 (18)
N5—H051⋯O2	0.83 (1)	2.62 (2)	3.1939 (12)	128 (1)
N5—H051⋯Cl1^ii^	0.83 (1)	2.69 (1)	3.3709 (9)	141 (1)
N5—H052⋯O2^iii^	0.85 (1)	2.08 (1)	2.8266 (11)	147 (2)
C4—H4*A*⋯N1^iv^	0.99	2.66	3.5236 (14)	146
C5—H05⋯Cl1^v^	0.95	2.82	3.6093 (10)	141

Symmetry codes: (i) 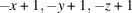; (ii) 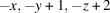; (iii) 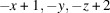; (iv) 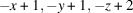; (v) 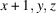.

**Table 3:** Experimental details

Crystal data	
Chemical formula	C_27_H_23_ClN_6_O_3_·C_3_H_7_NO
*M*_r_	588.06
Crystal system, space group	Triclinic, *P* 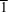
Temperature (K)	100
*a*, *b*, *c* (Å)	11.0172 (3), 12.3285 (3), 12.6748 (4)
α, β, γ (°)	64.450 (3), 89.344 (3), 78.658 (2)
*V* (Å^3^)	1517.63 (8)
*Z*	2
Radiation type	Mo *K*α
μ (mm^−1^)	0.18
Crystal size (mm)	0.16 × 0.16 × 0.12

Data collection	
Diffractometer	XtaLAB Synergy
Absorption correction	Multi-scan (*CrysAlis PRO*; Rigaku OD, 2025 ▸)
*T*_min_, *T*_max_	0.872, 1.000
No. of measured, independent and observed [*I* > 2σ(*I*)] reflections	100274, 11619, 9160
*R*_int_	0.056
θ values (°)	θ_max_ = 33.2, θ_min_ = 2.4
(sin θ/λ)_max_ (Å^−1^)	0.770

Refinement	
*R*[*F*^2^ > 2σ(*F*^2^)], *wR*(*F*^2^), *S*	0.050, 0.126, 1.07
No. of reflections	11619
No. of parameters	356
No. of restraints	6
H-atom treatment	H atoms treated by a mixture of independent and constrained refinement
Δρ_max_, Δρ_min_ (e Å^−3^)	0.56, −0.29

Computer programs: *CrysAlis PRO* (Rigaku OD, 2025 ▸), *SHELXT* (Sheldrick, 2015*a* ▸), *SHELXL2019/3* (Sheldrick, 2015*b* ▸), *XP* (Bruker, 1998 ▸) and *publCIF* (Westrip, 2010 ▸).

## full crystallographic data

### (E)-2,4-Diamino-5-{7-[(4-chlorophenyl)diazenyl]-3,3-dimethyl-1-oxo-2,3,4,9-tetrahydro-1H-xanthen-9-yl}-6-oxo-1,6-dihydropyridine-3-carbonitrile dimethylformamide monosolvate Crystal data

**Table:**

C_27_H_23_ClN_6_O_3_·C_3_H_7_NO	*Z* = 2
*M**_r_* = 588.06	*F*(000) = 696
Triclinic, *P*1	*D*_x_ = 1.447 Mg m^−^^3^
*a* = 11.0172 (3) Å	Mo *K*α radiation, λ = 0.71073 Å
*b* = 12.3285 (3) Å	Cell parameters from 38596 reflections
*c* = 12.6748 (4) Å	θ = 2.4–33.2°
α = 64.450 (3)°	µ = 0.18 mm^−^^1^
β = 89.344 (3)°	*T* = 100 K
γ = 78.658 (2)°	Irregular, orange
*V* = 1517.63 (8) Å^3^	0.16 × 0.16 × 0.12 mm

### (E)-2,4-Diamino-5-{7-[(4-chlorophenyl)diazenyl]-3,3-dimethyl-1-oxo-2,3,4,9-tetrahydro-1H-xanthen-9-yl}-6-oxo-1,6-dihydropyridine-3-carbonitrile dimethylformamide monosolvate Data collection

**Table:**

XtaLAB Synergy diffractometer	11619 independent reflections
Radiation source: micro-focus sealed X-ray tube	9160 reflections with *I* > 2σ(*I*)
Detector resolution: 10.0000 pixels mm^-1^	*R*_int_ = 0.056
ω scans	θ_max_ = 33.2°, θ_min_ = 2.4°
Absorption correction: multi-scan (CrysAlisPro; Rigaku OD, 2025)	*h* = −16→16
*T*_min_ = 0.872, *T*_max_ = 1.000	*k* = −18→18
100274 measured reflections	*l* = −19→19

### (E)-2,4-Diamino-5-{7-[(4-chlorophenyl)diazenyl]-3,3-dimethyl-1-oxo-2,3,4,9-tetrahydro-1H-xanthen-9-yl}-6-oxo-1,6-dihydropyridine-3-carbonitrile dimethylformamide monosolvate Refinement

**Table:**

Refinement on *F*^2^	Primary atom site location: dual
Least-squares matrix: full	Hydrogen site location: mixed
*R*[*F*^2^ > 2σ(*F*^2^)] = 0.050	H atoms treated by a mixture of independent and constrained refinement
*wR*(*F*^2^) = 0.126	*w* = 1/[σ^2^(*F*_o_^2^) + (0.0676*P*)^2^ + 0.2643*P*] where *P* = (*F*_o_^2^ + 2*F*_c_^2^)/3
*S* = 1.07	(Δ/σ)_max_ = 0.001
11619 reflections	Δρ_max_ = 0.56 e Å^−^^3^
356 parameters	Δρ_min_ = −0.29 e Å^−^^3^
6 restraints	

### (E)-2,4-Diamino-5-{7-[(4-chlorophenyl)diazenyl]-3,3-dimethyl-1-oxo-2,3,4,9-tetrahydro-1H-xanthen-9-yl}-6-oxo-1,6-dihydropyridine-3-carbonitrile dimethylformamide monosolvate Fractional atomic coordinates and isotropic or equivalent isotropic displacement parameters (Å^2^)

**Table:**

	*x*	*y*	*z*	*U*_iso_*/*U*_eq_	
N1	0.14828 (8)	0.65466 (8)	0.84932 (8)	0.02025 (17)	
N2	0.10393 (9)	0.76856 (9)	0.80198 (9)	0.02349 (18)	
O1	0.65579 (7)	0.52573 (6)	0.81948 (7)	0.01912 (14)	
C1	0.72160 (9)	0.18661 (9)	0.93834 (8)	0.01572 (16)	
O2	0.66692 (7)	0.10096 (7)	0.97332 (7)	0.02217 (15)	
C2	0.86182 (9)	0.16316 (9)	0.94458 (9)	0.01906 (18)	
H2A	0.893707	0.087344	0.934964	0.023*	
H2B	0.893364	0.148495	1.023451	0.023*	
C3	0.91361 (9)	0.26893 (10)	0.85173 (9)	0.01951 (18)	
C4	0.85135 (9)	0.38947 (9)	0.85687 (9)	0.01856 (18)	
H4A	0.885379	0.390258	0.928422	0.022*	
H4B	0.871513	0.459764	0.788082	0.022*	
C4A	0.71351 (9)	0.40505 (9)	0.85790 (8)	0.01557 (16)	
C5	0.47856 (10)	0.67623 (10)	0.80104 (11)	0.0235 (2)	
H05	0.530245	0.734582	0.776609	0.028*	
C6	0.35346 (10)	0.71337 (10)	0.80853 (10)	0.0231 (2)	
H06	0.318453	0.796859	0.790331	0.028*	
C7	0.27887 (9)	0.62564 (9)	0.84350 (9)	0.01862 (18)	
C8	0.33099 (9)	0.50313 (9)	0.87186 (8)	0.01639 (17)	
H08	0.279648	0.444474	0.897171	0.020*	
C8A	0.45709 (9)	0.46475 (8)	0.86387 (8)	0.01459 (16)	
C8B	0.52913 (9)	0.55330 (9)	0.82927 (9)	0.01740 (17)	
C9	0.51168 (8)	0.33465 (8)	0.88298 (8)	0.01328 (15)	
H9	0.485321	0.276919	0.958844	0.016*	
C9A	0.65145 (8)	0.31294 (8)	0.89250 (8)	0.01400 (15)	
N11	0.42705 (9)	0.37815 (8)	0.57725 (7)	0.01944 (17)	
H011	0.4374 (16)	0.4289 (16)	0.5031 (15)	0.036 (4)*	
C12	0.35524 (10)	0.29688 (10)	0.59099 (9)	0.01940 (18)	
C13	0.33734 (9)	0.21558 (9)	0.70441 (8)	0.01629 (17)	
C14	0.39335 (8)	0.22110 (8)	0.80298 (8)	0.01318 (15)	
C15	0.46177 (8)	0.31130 (8)	0.78490 (8)	0.01323 (15)	
C16	0.48166 (9)	0.38942 (9)	0.66910 (8)	0.01586 (16)	
N3	0.30702 (12)	0.29933 (11)	0.49221 (9)	0.0312 (2)	
H031	0.3275 (16)	0.3486 (15)	0.4255 (12)	0.040 (5)*	
H032	0.2477 (16)	0.2626 (18)	0.4947 (17)	0.058 (6)*	
N4	0.21380 (11)	0.04876 (11)	0.73639 (10)	0.0322 (2)	
N5	0.37648 (8)	0.14009 (8)	0.91206 (7)	0.01654 (15)	
H051	0.4246 (14)	0.1289 (15)	0.9669 (12)	0.030 (4)*	
H052	0.3407 (15)	0.0814 (14)	0.9237 (14)	0.037 (4)*	
O3	0.54610 (7)	0.47042 (7)	0.64269 (6)	0.02039 (15)	
C17	0.26884 (10)	0.12390 (10)	0.72123 (9)	0.02006 (19)	
C21	−0.02659 (9)	0.80208 (10)	0.80584 (10)	0.02030 (19)	
C22	−0.07321 (10)	0.92779 (10)	0.76723 (11)	0.0243 (2)	
H22	−0.018664	0.983383	0.740749	0.029*	
C23	−0.19902 (10)	0.97174 (10)	0.76743 (10)	0.0232 (2)	
H23	−0.231313	1.057128	0.742361	0.028*	
C24	−0.27692 (9)	0.88893 (9)	0.80484 (9)	0.01874 (18)	
Cl1	−0.43554 (2)	0.94466 (2)	0.79962 (2)	0.02262 (6)	
C25	−0.23163 (10)	0.76299 (10)	0.84473 (10)	0.0232 (2)	
H25	−0.286433	0.707667	0.871535	0.028*	
C26	−0.10597 (10)	0.71939 (10)	0.84486 (11)	0.0238 (2)	
H26	−0.073774	0.633779	0.871307	0.029*	
C27	1.05426 (10)	0.24386 (11)	0.87808 (12)	0.0273 (2)	
H27A	1.072060	0.240009	0.955343	0.041*	
H27B	1.087824	0.310192	0.818031	0.041*	
H27C	1.092951	0.165326	0.877734	0.041*	
C28	0.88616 (11)	0.27880 (11)	0.72925 (10)	0.0264 (2)	
H28A	0.924472	0.201162	0.726918	0.040*	
H28B	0.920329	0.345764	0.670810	0.040*	
H28C	0.796121	0.295779	0.711728	0.040*	

### (E)-2,4-Diamino-5-{7-[(4-chlorophenyl)diazenyl]-3,3-dimethyl-1-oxo-2,3,4,9-tetrahydro-1H-xanthen-9-yl}-6-oxo-1,6-dihydropyridine-3-carbonitrile dimethylformamide monosolvate Atomic displacement parameters (Å^2^)

**Table:**

	*U*^11^	*U*^22^	*U*^33^	*U*^12^	*U*^13^	*U*^23^
N1	0.0175 (4)	0.0204 (4)	0.0240 (4)	0.0002 (3)	−0.0005 (3)	−0.0125 (3)
N2	0.0173 (4)	0.0198 (4)	0.0350 (5)	0.0001 (3)	−0.0013 (3)	−0.0151 (4)
O1	0.0146 (3)	0.0128 (3)	0.0298 (4)	−0.0040 (2)	0.0003 (3)	−0.0087 (3)
C1	0.0161 (4)	0.0144 (4)	0.0149 (4)	−0.0032 (3)	−0.0003 (3)	−0.0047 (3)
O2	0.0199 (3)	0.0128 (3)	0.0274 (4)	−0.0048 (3)	−0.0011 (3)	−0.0023 (3)
C2	0.0153 (4)	0.0164 (4)	0.0236 (5)	−0.0018 (3)	−0.0010 (3)	−0.0076 (4)
C3	0.0152 (4)	0.0191 (4)	0.0255 (5)	−0.0044 (3)	0.0029 (3)	−0.0106 (4)
C4	0.0144 (4)	0.0173 (4)	0.0253 (5)	−0.0056 (3)	0.0018 (3)	−0.0096 (4)
C4A	0.0149 (4)	0.0134 (4)	0.0187 (4)	−0.0036 (3)	0.0008 (3)	−0.0070 (3)
C5	0.0186 (4)	0.0145 (4)	0.0394 (6)	−0.0037 (3)	−0.0034 (4)	−0.0135 (4)
C6	0.0192 (4)	0.0170 (4)	0.0358 (6)	−0.0011 (4)	−0.0041 (4)	−0.0151 (4)
C7	0.0164 (4)	0.0186 (4)	0.0224 (5)	−0.0010 (3)	−0.0012 (3)	−0.0116 (4)
C8	0.0153 (4)	0.0165 (4)	0.0181 (4)	−0.0033 (3)	0.0008 (3)	−0.0083 (3)
C8A	0.0149 (4)	0.0137 (4)	0.0157 (4)	−0.0031 (3)	0.0000 (3)	−0.0069 (3)
C8B	0.0147 (4)	0.0152 (4)	0.0234 (5)	−0.0032 (3)	−0.0012 (3)	−0.0094 (3)
C9	0.0138 (4)	0.0120 (4)	0.0136 (4)	−0.0040 (3)	0.0009 (3)	−0.0047 (3)
C9A	0.0140 (4)	0.0128 (4)	0.0145 (4)	−0.0037 (3)	0.0005 (3)	−0.0049 (3)
N11	0.0263 (4)	0.0199 (4)	0.0129 (4)	−0.0135 (3)	0.0018 (3)	−0.0043 (3)
C12	0.0239 (5)	0.0202 (4)	0.0168 (4)	−0.0107 (4)	0.0018 (3)	−0.0081 (3)
C13	0.0187 (4)	0.0149 (4)	0.0173 (4)	−0.0079 (3)	0.0019 (3)	−0.0071 (3)
C14	0.0126 (4)	0.0103 (3)	0.0158 (4)	−0.0028 (3)	0.0022 (3)	−0.0048 (3)
C15	0.0143 (4)	0.0115 (4)	0.0136 (4)	−0.0048 (3)	0.0015 (3)	−0.0044 (3)
C16	0.0181 (4)	0.0150 (4)	0.0144 (4)	−0.0064 (3)	0.0010 (3)	−0.0052 (3)
N3	0.0444 (6)	0.0377 (6)	0.0177 (4)	−0.0260 (5)	0.0010 (4)	−0.0107 (4)
N4	0.0406 (6)	0.0320 (5)	0.0342 (5)	−0.0231 (5)	0.0075 (4)	−0.0174 (4)
N5	0.0189 (4)	0.0137 (3)	0.0152 (4)	−0.0076 (3)	0.0016 (3)	−0.0029 (3)
O3	0.0268 (4)	0.0201 (3)	0.0152 (3)	−0.0151 (3)	0.0024 (3)	−0.0044 (3)
C17	0.0236 (5)	0.0199 (4)	0.0201 (4)	−0.0095 (4)	0.0030 (4)	−0.0099 (4)
C21	0.0165 (4)	0.0191 (4)	0.0271 (5)	−0.0003 (3)	−0.0016 (4)	−0.0131 (4)
C22	0.0210 (5)	0.0165 (4)	0.0341 (6)	−0.0032 (4)	0.0020 (4)	−0.0102 (4)
C23	0.0211 (5)	0.0139 (4)	0.0329 (5)	−0.0002 (3)	0.0001 (4)	−0.0101 (4)
C24	0.0155 (4)	0.0190 (4)	0.0224 (4)	0.0002 (3)	−0.0027 (3)	−0.0112 (4)
Cl1	0.01652 (11)	0.02239 (12)	0.02925 (13)	0.00158 (8)	−0.00234 (8)	−0.01387 (10)
C25	0.0180 (4)	0.0177 (4)	0.0338 (6)	−0.0025 (4)	−0.0037 (4)	−0.0116 (4)
C26	0.0189 (5)	0.0163 (4)	0.0369 (6)	0.0001 (4)	−0.0048 (4)	−0.0138 (4)
C27	0.0155 (4)	0.0281 (5)	0.0401 (6)	−0.0049 (4)	0.0051 (4)	−0.0166 (5)
C28	0.0288 (6)	0.0290 (5)	0.0258 (5)	−0.0089 (4)	0.0075 (4)	−0.0151 (4)

### (E)-2,4-Diamino-5-{7-[(4-chlorophenyl)diazenyl]-3,3-dimethyl-1-oxo-2,3,4,9-tetrahydro-1H-xanthen-9-yl}-6-oxo-1,6-dihydropyridine-3-carbonitrile dimethylformamide monosolvate Geometric parameters (Å, º)

**Table:**

N1—N2	1.2572 (13)	N11—C16	1.3875 (12)
N1—C7	1.4217 (13)	N11—H011	0.898 (17)
N2—C21	1.4224 (14)	C12—N3	1.3517 (14)
O1—C4A	1.3657 (12)	C12—C13	1.3923 (14)
O1—C8B	1.3871 (12)	C13—C17	1.4200 (13)
C1—O2	1.2306 (12)	C13—C14	1.4331 (13)
C1—C9A	1.4549 (13)	C14—N5	1.3489 (12)
C1—C2	1.5112 (14)	C14—C15	1.4011 (12)
C2—C3	1.5377 (14)	C15—C16	1.4094 (12)
C2—H2A	0.9900	C16—O3	1.2642 (11)
C2—H2B	0.9900	N3—H031	0.861 (13)
C3—C27	1.5306 (15)	N3—H032	0.858 (14)
C3—C28	1.5328 (16)	N4—C17	1.1522 (14)
C3—C4	1.5378 (14)	N5—H051	0.825 (12)
C4—C4A	1.4933 (13)	N5—H052	0.848 (13)
C4—H4A	0.9900	C21—C22	1.3941 (15)
C4—H4B	0.9900	C21—C26	1.3980 (15)
C4A—C9A	1.3485 (13)	C22—C23	1.3859 (15)
C5—C6	1.3800 (15)	C22—H22	0.9500
C5—C8B	1.3928 (14)	C23—C24	1.3861 (15)
C5—H05	0.9500	C23—H23	0.9500
C6—C7	1.4033 (15)	C24—C25	1.3926 (14)
C6—H06	0.9500	C24—Cl1	1.7383 (10)
C7—C8	1.3911 (14)	C25—C26	1.3828 (15)
C8—C8A	1.3942 (13)	C25—H25	0.9500
C8—H08	0.9500	C26—H26	0.9500
C8A—C8B	1.3907 (13)	C27—H27A	0.9800
C8A—C9	1.5107 (13)	C27—H27B	0.9800
C9—C9A	1.5072 (13)	C27—H27C	0.9800
C9—C15	1.5223 (13)	C28—H28A	0.9800
C9—H9	1.0000	C28—H28B	0.9800
N11—C12	1.3483 (13)	C28—H28C	0.9800
			
N2—N1—C7	112.74 (9)	C12—N11—H011	116.4 (11)
N1—N2—C21	114.76 (9)	C16—N11—H011	119.3 (11)
C4A—O1—C8B	118.26 (7)	N11—C12—N3	116.93 (9)
O2—C1—C9A	120.10 (9)	N11—C12—C13	118.49 (9)
O2—C1—C2	121.11 (9)	N3—C12—C13	124.56 (9)
C9A—C1—C2	118.77 (8)	C12—C13—C17	119.55 (9)
C1—C2—C3	114.03 (8)	C12—C13—C14	119.79 (8)
C1—C2—H2A	108.7	C17—C13—C14	120.59 (8)
C3—C2—H2A	108.7	N5—C14—C15	121.31 (8)
C1—C2—H2B	108.7	N5—C14—C13	118.77 (8)
C3—C2—H2B	108.7	C15—C14—C13	119.90 (8)
H2A—C2—H2B	107.6	C14—C15—C16	118.79 (8)
C27—C3—C28	109.25 (9)	C14—C15—C9	123.90 (8)
C27—C3—C2	109.27 (9)	C16—C15—C9	117.25 (8)
C28—C3—C2	110.06 (9)	O3—C16—N11	117.22 (8)
C27—C3—C4	109.92 (9)	O3—C16—C15	124.23 (9)
C28—C3—C4	109.77 (9)	N11—C16—C15	118.55 (8)
C2—C3—C4	108.56 (8)	C12—N3—H031	118.6 (12)
C4A—C4—C3	112.29 (8)	C12—N3—H032	120.6 (13)
C4A—C4—H4A	109.1	H031—N3—H032	119.9 (17)
C3—C4—H4A	109.1	C14—N5—H051	118.2 (11)
C4A—C4—H4B	109.1	C14—N5—H052	121.7 (11)
C3—C4—H4B	109.1	H051—N5—H052	115.4 (16)
H4A—C4—H4B	107.9	N4—C17—C13	179.12 (12)
C9A—C4A—O1	123.10 (9)	C22—C21—C26	120.48 (10)
C9A—C4A—C4	125.22 (9)	C22—C21—N2	114.69 (9)
O1—C4A—C4	111.67 (8)	C26—C21—N2	124.83 (9)
C6—C5—C8B	120.08 (10)	C23—C22—C21	120.08 (10)
C6—C5—H05	120.0	C23—C22—H22	120.0
C8B—C5—H05	120.0	C21—C22—H22	120.0
C5—C6—C7	118.84 (10)	C22—C23—C24	118.84 (10)
C5—C6—H06	120.6	C22—C23—H23	120.6
C7—C6—H06	120.6	C24—C23—H23	120.6
C8—C7—C6	120.34 (9)	C23—C24—C25	121.77 (10)
C8—C7—N1	116.11 (9)	C23—C24—Cl1	118.85 (8)
C6—C7—N1	123.54 (9)	C25—C24—Cl1	119.37 (8)
C7—C8—C8A	121.30 (9)	C26—C25—C24	119.23 (10)
C7—C8—H08	119.4	C26—C25—H25	120.4
C8A—C8—H08	119.4	C24—C25—H25	120.4
C8B—C8A—C8	117.29 (9)	C25—C26—C21	119.59 (10)
C8B—C8A—C9	121.08 (8)	C25—C26—H26	120.2
C8—C8A—C9	121.47 (8)	C21—C26—H26	120.2
O1—C8B—C8A	122.45 (9)	C3—C27—H27A	109.5
O1—C8B—C5	115.40 (8)	C3—C27—H27B	109.5
C8A—C8B—C5	122.14 (9)	H27A—C27—H27B	109.5
C9A—C9—C8A	109.52 (7)	C3—C27—H27C	109.5
C9A—C9—C15	112.34 (7)	H27A—C27—H27C	109.5
C8A—C9—C15	110.07 (7)	H27B—C27—H27C	109.5
C9A—C9—H9	108.3	C3—C28—H28A	109.5
C8A—C9—H9	108.3	C3—C28—H28B	109.5
C15—C9—H9	108.3	H28A—C28—H28B	109.5
C4A—C9A—C1	119.03 (8)	C3—C28—H28C	109.5
C4A—C9A—C9	123.00 (8)	H28A—C28—H28C	109.5
C1—C9A—C9	117.95 (8)	H28B—C28—H28C	109.5
C12—N11—C16	124.32 (8)		
			
C7—N1—N2—C21	−179.90 (8)	C2—C1—C9A—C9	177.87 (8)
O2—C1—C2—C3	153.20 (10)	C8A—C9—C9A—C4A	−16.02 (12)
C9A—C1—C2—C3	−28.24 (13)	C15—C9—C9A—C4A	106.62 (10)
C1—C2—C3—C27	171.41 (9)	C8A—C9—C9A—C1	165.67 (8)
C1—C2—C3—C28	−68.62 (11)	C15—C9—C9A—C1	−71.68 (10)
C1—C2—C3—C4	51.54 (11)	C16—N11—C12—N3	178.74 (11)
C27—C3—C4—C4A	−167.54 (9)	C16—N11—C12—C13	−2.26 (17)
C28—C3—C4—C4A	72.27 (11)	N11—C12—C13—C17	−175.92 (10)
C2—C3—C4—C4A	−48.07 (11)	N3—C12—C13—C17	2.99 (18)
C8B—O1—C4A—C9A	7.66 (14)	N11—C12—C13—C14	0.93 (15)
C8B—O1—C4A—C4	−172.08 (8)	N3—C12—C13—C14	179.84 (11)
C3—C4—C4A—C9A	22.81 (14)	C12—C13—C14—N5	−178.89 (9)
C3—C4—C4A—O1	−157.45 (8)	C17—C13—C14—N5	−2.08 (14)
C8B—C5—C6—C7	−0.76 (17)	C12—C13—C14—C15	2.50 (14)
C5—C6—C7—C8	1.04 (16)	C17—C13—C14—C15	179.31 (9)
C5—C6—C7—N1	−177.21 (10)	N5—C14—C15—C16	176.81 (9)
N2—N1—C7—C8	−167.33 (9)	C13—C14—C15—C16	−4.62 (14)
N2—N1—C7—C6	10.98 (15)	N5—C14—C15—C9	−6.13 (14)
C6—C7—C8—C8A	−1.49 (15)	C13—C14—C15—C9	172.43 (8)
N1—C7—C8—C8A	176.89 (9)	C9A—C9—C15—C14	118.20 (10)
C7—C8—C8A—C8B	1.58 (14)	C8A—C9—C15—C14	−119.47 (10)
C7—C8—C8A—C9	−173.89 (9)	C9A—C9—C15—C16	−64.70 (11)
C4A—O1—C8B—C8A	−7.51 (14)	C8A—C9—C15—C16	57.63 (11)
C4A—O1—C8B—C5	173.03 (9)	C12—N11—C16—O3	−179.59 (10)
C8—C8A—C8B—O1	179.27 (9)	C12—N11—C16—C15	0.09 (16)
C9—C8A—C8B—O1	−5.24 (14)	C14—C15—C16—O3	−176.96 (9)
C8—C8A—C8B—C5	−1.31 (15)	C9—C15—C16—O3	5.79 (15)
C9—C8A—C8B—C5	174.18 (9)	C14—C15—C16—N11	3.38 (14)
C6—C5—C8B—O1	−179.61 (10)	C9—C15—C16—N11	−173.87 (9)
C6—C5—C8B—C8A	0.93 (17)	N1—N2—C21—C22	172.06 (10)
C8B—C8A—C9—C9A	15.75 (12)	N1—N2—C21—C26	−7.68 (16)
C8—C8A—C9—C9A	−168.95 (8)	C26—C21—C22—C23	0.09 (17)
C8B—C8A—C9—C15	−108.23 (10)	N2—C21—C22—C23	−179.66 (10)
C8—C8A—C9—C15	67.07 (11)	C21—C22—C23—C24	−0.95 (17)
O1—C4A—C9A—C1	−176.54 (9)	C22—C23—C24—C25	1.60 (17)
C4—C4A—C9A—C1	3.17 (15)	C22—C23—C24—Cl1	−177.48 (9)
O1—C4A—C9A—C9	5.17 (15)	C23—C24—C25—C26	−1.34 (17)
C4—C4A—C9A—C9	−175.12 (9)	Cl1—C24—C25—C26	177.73 (9)
O2—C1—C9A—C4A	178.07 (9)	C24—C25—C26—C21	0.43 (17)
C2—C1—C9A—C4A	−0.51 (13)	C22—C21—C26—C25	0.18 (17)
O2—C1—C9A—C9	−3.56 (13)	N2—C21—C26—C25	179.90 (11)

### (E)-2,4-Diamino-5-{7-[(4-chlorophenyl)diazenyl]-3,3-dimethyl-1-oxo-2,3,4,9-tetrahydro-1H-xanthen-9-yl}-6-oxo-1,6-dihydropyridine-3-carbonitrile dimethylformamide monosolvate Hydrogen-bond geometry (Å, º)

**Table:**

*D*—H···*A*	*D*—H	H···*A*	*D*···*A*	*D*—H···*A*
N11—H011···O3^i^	0.898 (17)	1.758 (17)	2.6558 (11)	178.7 (18)
N5—H051···O2	0.83 (1)	2.62 (2)	3.1939 (12)	128 (1)
N5—H051···Cl1^ii^	0.83 (1)	2.69 (1)	3.3709 (9)	141 (1)
N5—H052···O2^iii^	0.85 (1)	2.08 (1)	2.8266 (11)	147 (2)
C4—H4*A*···N1^iv^	0.99	2.66	3.5236 (14)	146
C5—H05···Cl1^v^	0.95	2.82	3.6093 (10)	141

Symmetry codes: (i) −*x*+1, −*y*+1, −*z*+1; (ii) −*x*, −*y*+1, −*z*+2; (iii) −*x*+1, −*y*, −*z*+2; (iv) −*x*+1, −*y*+1, −*z*+2; (v) *x*+1, *y*, *z*.
